# Collaborative learning health systems: Science and practice

**DOI:** 10.1002/lrh2.10286

**Published:** 2021-07-08

**Authors:** David M. Hartley, Michael Seid

**Affiliations:** ^1^ James M. Anderson Center for Health Systems Excellence Cincinnati Children's Hospital Cincinnati Ohio USA; ^2^ Department of Pediatrics University of Cincinnati, College of Medicine Cincinnati Ohio USA; ^3^ Division of Pulmonary Medicine Cincinnati Children's Hospital Cincinnati Ohio USA

Improving the U.S. healthcare system to achieve better health outcomes for all is one of the most pressing public health challenges of our time. A Learning Health System ‐ in which clinical care, science, informatics, incentives, and culture are aligned for continuous improvement, innovation, and research; new knowledge is captured as a by‐product of care; and evidence is applied reliably and seamlessly embedded in the delivery process ‐ is one promising response to this challenge.^1^ A subset of Learning Health Systems is Collaborative Learning Health Systems (CLHSs), also called Learning Health Networks, which use a network organizational architecture to facilitate collaboration at scale to improve health outcomes. CLHSs exist for many conditions, and more are under development. Many have demonstrated substantial improvements in outcomes and, as such, are models for healthcare transformation, providing important opportunities for study and learning. For this reason, CLHSs are the focus of this special issue of Learning Health Systems.

Each work in this issue is a self‐contained study conveying important messages and advancing the field, but they fit together to create a bigger whole. *Seid* et al. (“Science of collaborative learning health systems”) offer a framing for CLHSs as complex adaptive systems in which communities of all stakeholder types are able to collaborate, at scale, to create, and share resources to satisfy a variety of needs. A key insight is that the infrastructure and services underlying a CLHS are designed to enable stakeholders to act on their inherent motivations. This is a frameshift from a model that focuses on changing people in the system to one that focuses on adapting the system to better enable people to do what they need to do. Collaborating at scale implies new ways of interacting within the community. *Vinson* explores implications of conceptualizing culture ‐ the systems of social relations, meanings, and forms of expression shared among group members ‐ as infrastructure in learning health systems. Her perspective describes important organizational and behavioral aspects of CLHSs, “peopling” the system.

The centrality of culture and people stand out clearly in the work of *Thygeson* et al., which documents the impact of a relational intervention on participant engagement, self‐efficacy, and motivation as well as spontaneous, emergent dissemination of relational change and learning to other parts of a health system. On a wider scale, *Keck* et al. describe efforts to create conditions for the production and sharing of information, knowledge, and know‐how so that more people in a CLHS can get “what is needed, when it's needed.” A key insight here is that system‐level interventions (community organizing, digital outreach) enable individual‐level problem solving (accessing and using resources created by the community). In the case of CLHSs focused on pediatric conditions, the community includes young people. *David* et al. illustrate the benefits of integrating these youth into the learning health system as experts developing patient‐generated resources in a sustainable manner. Integrating all participants into the CLHS is a goal, but efforts can fall short, resulting in inequity in outcomes. *Parsons* et al. offer a set of core practices to achieve and sustain equity in learning health systems, as well as case examples of this deeply complex and challenging (at an individual, institutional, and structural level) work. *Wood* et al. expand this to consider the ways in which learning health systems might be a way to instantiate the idea of socially accountable health professional education. *Schleyer* et al. provide a detailed description of the establishment of a statewide, inter‐organizational CLHS. Their work demonstrates the importance of governance decisions, shared goal setting and monitoring, non‐siloed information exchange, and project selection to success.

As these works illustrate, there is a large set of needs and interventions in the development and optimization of CLHSs. Often, it's not clear what to do, and relying on experiential and experimental learning is too slow. The research report of *Seid* et al. (“Collaborative learning health system agent‐based model…”) provides a potential approach to accelerate learning. Their demonstration of the computational and face validity of a learning health system agent‐based model provides a glimpse of a way to simulate the effects of (sets of) interventions on CLHSs, with implications for much more rapid and efficient learning and scaling. Indeed, as analogous to a mouse model for clinical research, this CLHS model points a way towards thinking of a spectrum of CLHS science.

Such a spectrum can be categorized in a way similar to the schema articulated by the National Center for Advancing Translational Sciences (NCATS) and the Agency for Healthcare Research and Quality (AHRQ). NCATS describes a translational science spectrum including basic research, preclinical research, clinical research, clinical implementation, and public health. Similarly, AHRQ defines categories of research needed for transforming the healthcare system, including those investigating basic to clinical efficacy (called “T1” research), clinical efficacy to clinical effectiveness (T2), and effectiveness to outcomes (T3). If we imagine the logical extension of this sequence, a category of outcomes to population health (T4) might be added. Such categories have implications for organizing research around scientific endeavors that increase our understanding of CLHSs, using these to develop and test interventions, and ensuring that CLHSs fulfill their promise to improve health for all. In that vein, the collection of works in this issue fall throughout this continuum, spanning fundamental and applied research to implementation:

- Works concerned with basic and fundamental questions (T0). Such research includes description and observation, natural history, measurement, modeling, and mechanisms of action. Studies falling in this category include *Seid* et al. (“Science of collaborative learning health systems”), *Vinson*, and *Seid* et al. (“Collaborative learning health system agent‐based model…”).
- Studies concerned with translational research (T1‐T3). Such research includes experiments conducted in a structured and predictable setting to better understand process or effect. Studies falling in this category include *Thygeson* et al., *Keck* et al., and *David* et al.
- Studies concerned with implementation research (T4). These studies test hypotheses in real‐world settings, adjust for context and setting, apply quality improvement methods, and assess/improve outcomes. Studies falling in this category include *Parsons* et al., *Wood* et al., and *Schleyer* et al.

The collected articles in this Special Issue highlight the balance between science and practice and illustrate how CLHS science is a transdisciplinary endeavor involving medicine, epidemiology, social and political science, organization behavior, and informatics among other fields. This compilation of work is valuable not only as a statement of the why, what, and how of CLHSs, but also as a foundation for a scientific agenda for further understanding and improving them.
